# A missense variant in the nuclear localization signal of DKC1 causes Hoyeraal-Hreidarsson syndrome

**DOI:** 10.1038/s41525-022-00335-8

**Published:** 2022-10-30

**Authors:** Chia-Mei Chu, Hsin-Hui Yu, Tsai-Ling Kao, Yi-Hsuan Chen, Hsuan-Hsuan Lu, En-Ting Wu, Yun-Li Yang, Chin-Hsien Lin, Shin-Yu Lin, Meng-Ju Melody Tsai, Yin-Hsiu Chien, Wuh-Liang Hwu, Wen-Pin Chen, Ni-Chung Lee, Chi-Kang Tseng

**Affiliations:** 1 Department of Microbiology, College of Medicine, National Taiwan University, Taipei, Taiwan; 2 Department of Pediatrics National Taiwan University Children’s Hospital Taipei, Taipei, Taiwan; 3 Center for Frontier Medicine, National Taiwan University Hospital, Taipei, Taiwan; 4 Department of Neurology, National Taiwan University Hospital, Taipei, Taiwan; 5 Depatment of Obstetrics and Gynecology, National Taiwan University Hospital, Taipei, Taiwan; 6 Department of Medical Genetics, National Taiwan University Hospital, Taipei, Taiwan; 7 Institute of Pharmacology, College of Medicine, National Taiwan University, Taipei City, Taiwan

**Keywords:** Diseases, Genomic instability

## Abstract

Hoyeraal-Hreidarsson syndrome (HHS) is the most severe form of dyskeratosis congenita (DC) and is caused by mutations in genes involved in telomere maintenance. Here, we identified male siblings from a family with HHS carrying a hemizygous mutation (c.1345C > G, p.R449G), located in the C-terminal nuclear localization signal (NLS) of the *DKC1* gene. These patients exhibit progressive cerebellar hypoplasia, recurrent infections, pancytopenia due to bone marrow failure, and short leukocyte telomere lengths. Single-cell RNA sequencing analysis suggested defects in the NLRP3 inflammasome in monocytes and the activation and maturation of NK cells and B cells. In experiments using induced pluripotent stem cells (iPSCs) from patients, DKC1_R449G iPSCs had short telomere lengths due to reduced levels of human telomerase RNA (hTR) and increased cytosolic proportions of DKC1. Treatment with dihydroquinolizinone RG7834 and 3′deoxyanosine cordycepin rescued telomere length in patient-derived iPSCs. Together, our findings not only provide new insights into immunodeficiency in DC patients but also provide treatment options for telomerase insufficiency disorders.

## Introduction

*DKC1*, which encodes a nucleolar protein called DKC1 (dyskerin)^1^, is the first gene identified to play a role in dyskeratosis congenita (DC), which is a rare inherited disease that is estimated to affect approximately one person per million people and is mainly characterized by the triad of abnormal skin pigmentation, oral leucoplakia, and nail dystrophy^2^. Hoyeraal-Hreidarsson syndrome (HHS) is the most severe form of DC and has a very early age of onset^3,4^. Patients with HHS are characterized by very short telomeres and manifest additional features compared to DC patients, such as cerebellar hypoplasia^3,4^. At the molecular level, DC/HHS patients have characteristic accelerated telomere shortening^2,5^. Recurrent infection is frequently seen in DC/HH patients due to progressive bone marrow failure with compromised immune function^2^. In addition, the mode of inheritance is significantly associated with overall survival. The X-linked recessive disorders have a higher cancer risk and the worst survival, compared with autosomal dominant disorders^6^. A therapeutic strategy for telomere disorder-related diseases remains to be developed.

## Results

### Clinical phenotype and genetic characterization of a family with HHS

**Fig. 1:**
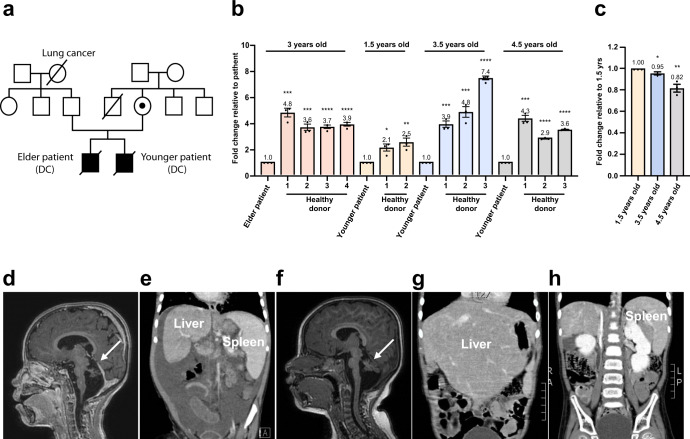
Clinical phenotype and telomere length measurement. **a** Pedigree of the family. Unaffected individuals are shown as open shapes. The carrier is shown as a half-filled shape. Clinically affected individuals are shown as filled shapes. A deceased individual is shown as a shape with a diagonal line. Squares represent males. Circles represent females. **b** Telomere length measurement by quantitative PCR (qPCR) of peripheral blood cell genomic DNA from patients in the family and age-matched controls. Autosomal single copy gene (*36B4*) served as the basis for normalizing the telomere quantitative PCR signal. Bar graph of mean fold change for telomere length of age-matched samples relative to patients. Mean values were calculated from triplicate qRT-PCR experiments of three technical replicates, with bars representing SE. **c** Telomere length measurement by quantitative PCR (qPCR) of peripheral blood cell genomic DNA from the younger patient in the family. Autosomal single copy gene (*36B4*) served as the basis for normalizing the telomere quantitative PCR signal. Bar graph of mean fold change for telomere length of samples as indicated age relative to the sample from 1.5 years old. Mean values were calculated from triplicate qRT-PCR experiments of three technical replicates, with bars representing standard error of the mean (s.e.m.). Brain MRI showing cerebellar hypoplasia in the elder patient at 2.5 years old (**d**) and the younger patient at 1 year old (**f**). An abdominal computed tomography scan revealed hepatosplenomegaly in the elder brother at 1.3 years old (**e**) and in the younger brother at 3 years old (**g** and **h**).

We identified siblings from a family with HHS. Whole-exome sequencing (WES) of the patients identified a hemizygous c.1345C > G (p.R449G) variant in the *DKC1* gene (NM_001363) (Supplementary Fig. 1a). This missense variant was considered to be disease-causing (ClinVar variation ID: 235576), with an ACMG classification of likely pathogenic (PS3 PM2 PP1 PP2 PP3 PP4). Sanger sequencing confirmed that the elder and younger patients had the same hemizygous variant, while the mother was a carrier (Fig. 1a and Supplementary Fig. 1b). The patient’s mother had a brother who died at 3 years of age for unknown reasons (Fig. 1a). The telomere lengths in gDNA from circulating leukocytes of the siblings were shorter than those of age-matched controls in a quantitative PCR assay (Fig. 1b). In addition, the telomere lengths of younger brother shortened with time (Fig. 1c).

The elder patient presented with intrauterine growth restriction, developmental delay, cerebellar hypoplasia (Fig. 1d), and hepatosplenomegaly (Fig. 1e) since the ages of 1 year and 4 months. Severe aplastic anaemia with progressive pancytopenia developed at the ages of 2 years and 6 months. He died at 3 years old due to *Burkholderia cenocepacia* bacteremia and *Pneumocystis jirovec*i pneumonia (PJP) complicated with severe acute respiratory distress syndrome (ARDS) (Supplementary Table 1). The younger patient manifested developmental delay, ataxia, cerebellar hypoplasia (Fig. 1f), hepatosplenomegaly (Fig. 1g, h), progressive leukopenia, lymphopenia, and thrombocytopenia since he was 1.6 years of age, and anaemia and leukopenia since he was 3.5 years of age. He had recurrent viral (adenovirus pneumonia with severe ARDS, herpes simplex virus), bacterial (*Acinetobacter baumannii*), and fungal (PJP, *Candida albicans*) infections since he was 2.8 years old despite regular intravenous immunoglobulin (IVIG) administration and prophylactic antimicrobial agent usage. Unfortunately, there was no chance to perform hematopoietic stem cell transplantation under stable conditions. He died at 5.7 years of age due to PJP with respiratory failure.

### Single-cell RNA-seq of PBMCs showing immunodeficiency in the DC patient

**Fig. 2:**
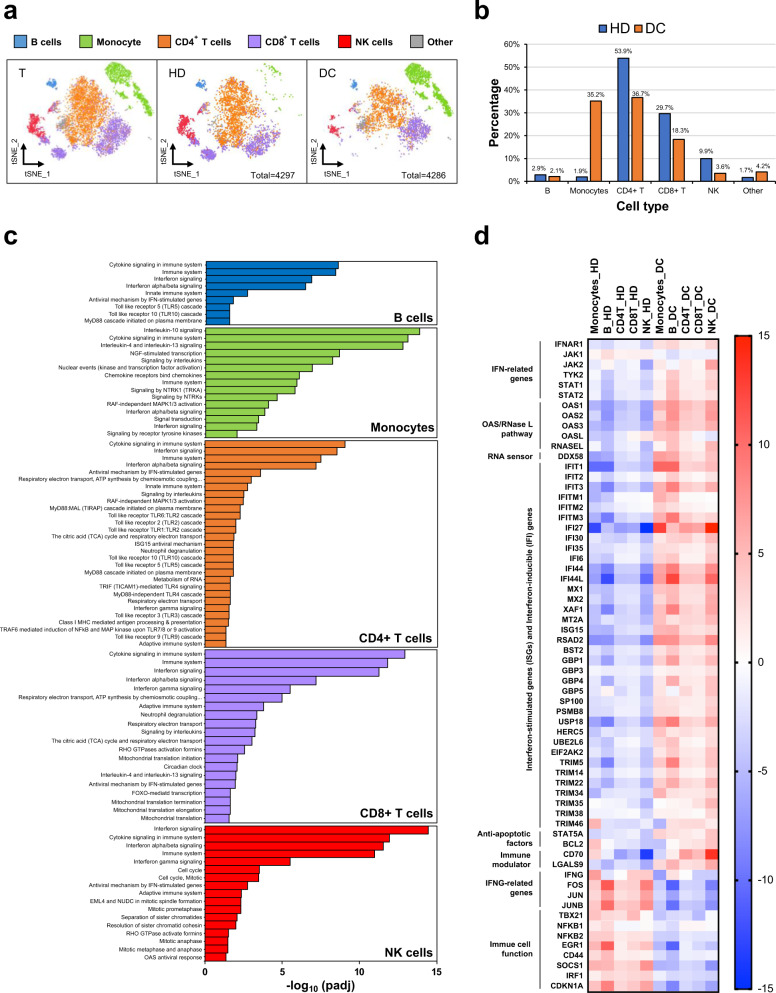
Single-cell RNA-seq of PBMCs showing immunodeficiency in the DC patient. **a** t-distributed stochastic neighbour embedding (tSNE) analysis of PBMCs from the healthy donor (HD) and DC patient. A total of 4297 and 4286 single cells for the HD and DC subjects were clustered into five major clusters (B cells, CD4+T cells, CD8+T cells, monocytes, and NK cells). Cells were annotated by independently correlating each cell with reference transcriptomic datasets. T, Totality of cells analysed; HD, healthy donor; DC, DC patient. **b** Bar graph showing the percentage of each cell type relative to the total cell numbers in the healthy donor or DC patient. **c** Enriched pathways in the DC patient by Reactome analysis in each immune cell subset. **d** Heatmap of anti-pathogen response genes in immune cells for the healthy donor and DC patient coloured by average fold change.

Immunological studies for the younger patient revealed decreased T, B, and NK lymphocytes, normal IgG and IgA levels, and transient elevation of IgM levels (Supplementary Table 1). To dissect complex immune cell functions in healthy individuals and DCs, single-cell RNA sequencing (scRNA-seq) was performed on PBMCs from the younger DC patient (DC) and the age-matched healthy donor (HD) (Fig. 2a). Five major clusters (B cells, CD4^+^ T cells, CD8^+^ T cells, monocytes, and NK cells) were clustered based on known cell-type-specific gene profiles^7^ (Fig. 2a). The results indicated that the size of the monocyte population was increased. However, there were reductions in the proportions of B cells, CD4^+^ T cells, CD8^+^ T cells, and NK cells (Fig. 2b).

An integrated analysis of PBMCs was performed. Reactome analysis indicated that immune system- and anti-pathogen-related pathways were enriched (Fig. 2c). Genes involved in the anti-microbial response were upregulated, including IFN-related genes (*IFNAR1, JAK1/2, TYK2, STAT1/2*), genes involved in the OAS/RNase L pathway (*OAS1, PAS2, OAS3, OASL, and RNase L*), and the RNA sensor DDX58 (*RIG-I*) (Fig. 2d). Additionally, interferon-stimulated genes (ISGs) and interferon-inducible (IFI) genes were upregulated (Fig. 2d). The expression of anti-apoptotic survival factors, such as *STAT5A* and *BCL-2*, was increased (Fig. 2d). Expression of the pleiotropic immune modulator *LGALS9*, which encodes Galectin-9 (Gal-9), was increased in all cells^8^ (Fig. 2d).

The expression levels of IFN-γ (*IFNG*) and genes involved in the expression of IFN-γ (*FOS, JUN, TBX21*) were reduced (Fig. 2d). IFN-γ is a critical cytokine that activates macrophages and links the innate and adaptive immune responses^9^. We also observed reductions in the expression levels of NF-κB genes (*NFKB1 and NFKB2*), which are crucial regulators of inducible gene expression involved in the development and function of immune cells^10^. Overall, the integrated analysis of single-cell RNA-seq data from PBMCs in combination with recurrent and opportunistic infections at initial presentation suggested that the immune system of the DC patient was dysfunctional.

### Single-cell RNA-Seq analysis reveals distinct contributions of monocyte and NK-cell populations in the DC patient

**Fig. 3:**
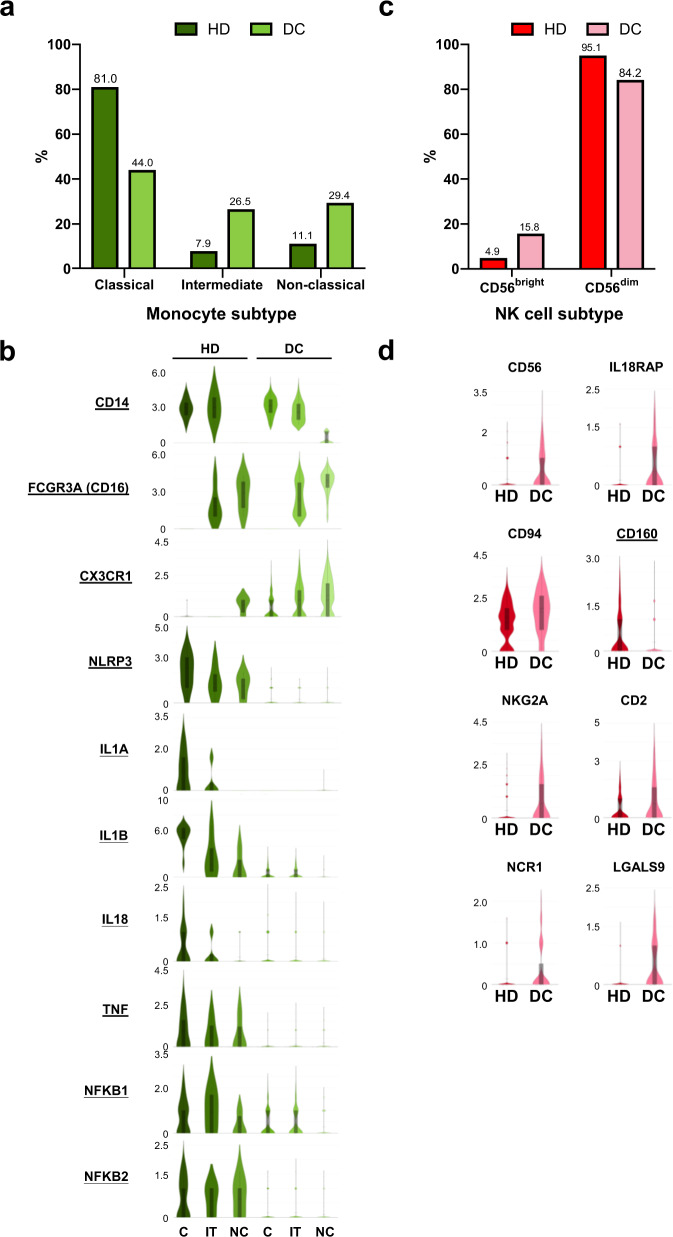
Single-cell RNA-Seq analysis reveals distinct contributions of monocyte and NK-cell populations in the DC patient. **a** Bar graph showing proportions of distinct monocyte subsets relative to their total monocyte counts in the healthy donor and DC patient, respectively. **b** Violin plots showing the differential expression distribution of monocyte-associated genes between the healthy donor and DC patient in classical, intermediate, and nonclassical monocytes. The box represents the interquartile range; The whiskers represent the rest of the distribution. **c** Bar graph showing the proportions of the CD56^bright^ and CD56^dim^ subsets in the healthy donor and DC patient. **d** Violin plots showing the differential expression distribution of NK-cell-associated genes between the healthy donor and DC patient. The box represents the interquartile range; The whiskers represent the rest of the distribution.

A large proportion of monocytes was observed in the DC patient (Fig. 2b). To examine the function of monocytes, three distinct monocyte subsets were grouped according to the expression of CD14 and CD16: classical monocytes (CD14^hi^CD16^**−**^), intermediate monocytes (CD14^hi^CD16^+^), and nonclassical monocytes (CD14^Low^CD16^hi^) (Fig. 3a). Monocytes egress from the bone marrow as classical monocytes (CD14^hi^CD16^**−**^), and a portion of them subsequently differentiate into intermediate monocytes (CD14^hi^CD16^+^) and nonclassical monocytes (CD14^Low^CD16^hi^)^11^. Nonclassical monocytes have been suggested to represent an aged monocyte subset due to detection of several ageing markers, such as short telomere length^12^ and high expression levels of CX3CR1^13^. Since the DC patient exhibited shorter telomeres than the age-matched control (Fig. 1b), we examined the monocyte ageing marker CX3CR1. CX3CR1 was highly expressed on three types of monocytes from the DC patient (Fig. 3b), suggesting that monocytes derived from the DC patient prematurely undergo the ageing process.

In contrast to nonclassical monocytes, classical monocytes exhibit high expression of genes involved in responses to bacterial infection and inflammation and genes involved in inflammasome signalling^14^. In the DC patient, reductions in the proportion of classical monocytes (Fig. 3a) and the expression levels of NF-κB (Fig. 2d), which is a transcription factor for many proinflammatory cytokines, prompted us to speculate that proinflammatory cytokine production/secretion was impaired in the DC patient. As expected, the expression levels of genes involved in NLRP3 inflammasome formation^15^ (*NLRP3*, *IL1β*, *IL-18*, *TNF*, *NFKB1*, and *NFKB2*) were dramatically decreased (Fig. 3b). The NLRP3 inflammasome triggers caspase-1 activation and secretion of the proinflammatory cytokines IL-1β and IL-18^16^. IL-18 is essential for IFN-γ production, which augments the cytotoxicity of NK cells and T cells^17^. Low expression levels of NLRP3 inflammasome-related genes (Fig. 3b) and IFN-γ (Fig. 2d) suggest that downstream immune responses are defective.

We grouped NK-cell subsets. The proportion of immature CD56^bright^ NK cells, which normally represent a maximum of 10% of all peripheral blood NK cells, was increased (15.8%) concomitant with a decrease in the proportion of CD56^dim^ NK cells (84.2%) (Fig. 3c). The DC patient displayed high expression levels of CD56, the major inhibitory receptor (*CD94* and *NKG2A*), the activating receptor NCR1 (*NKp46*), the cytokine receptor *IL18RAP*, and the adhesion molecule CD2 but low expression levels of *CD160*, which is mainly expressed by CD56^dim^ NK cells^18^. The expression levels of *LGALS9*, which has been shown to impair NK-cell functions by affecting cell-mediated cytotoxicity and decreasing IFN-γ production, were increased^19^ (Fig. 3d).

### The DC patient exhibits defects in B-cell function

**Fig. 4:**
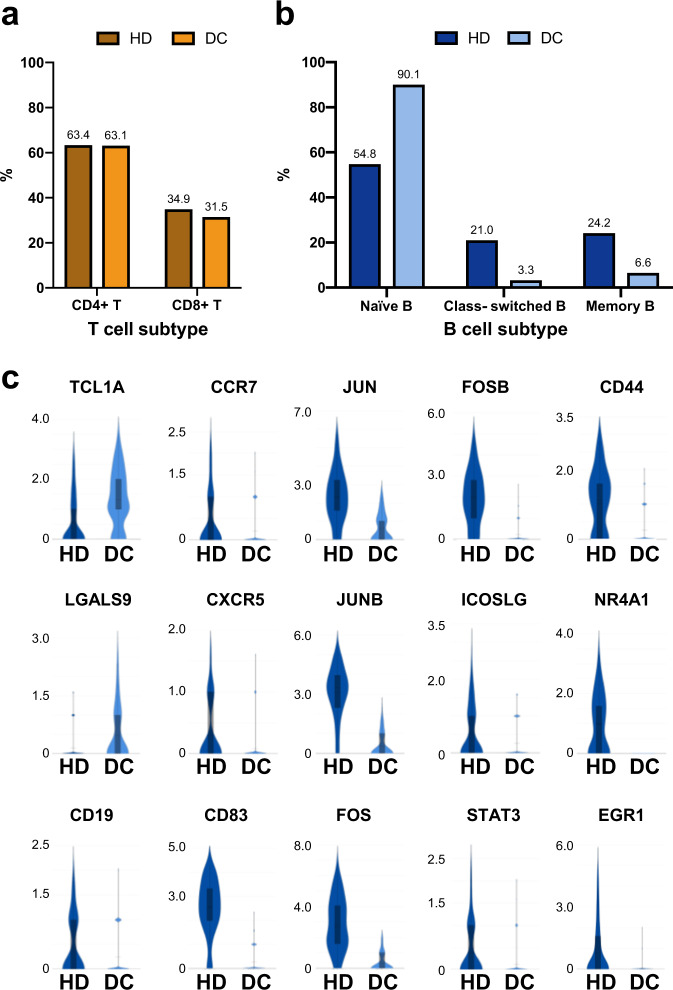
The DC patient exhibits defects in B-cell function. **a** Bar graph showing CD4^+^ T and CD8^+^ T-cell proportions in the healthy donor and DC patient. **b** Bar graph showing distinct B-cell subset proportions in the healthy donor and DC patient. **c** Violin plots showing the differential expression levels of B-cell marker genes in B cells between the healthy donor and DC patient. The box represents the interquartile range; The whiskers represent the rest of the distribution.

T- and B-cell immunodeficiency was the predominant finding in the DC patients at initial presentation (Supplementary Table 1). Next, we analysed T and B cells in DC samples. The proportions of CD4^+^ and CD8^+^ T cells were not substantially different between the healthy donor and DC patient (Fig. 4a). Additionally, the expression of genes encoding exhaustion markers (*PD-1*, *LAG3*, *CTLA-4*, *TIGIT*, *KLRG1*) on T cells was not significantly different. However, the DC patient showed a high percentage of naïve B cells (90%) (Fig. 4b). Supporting this observation, B cells from the DC patient showed high expression levels of a naïve B-cell marker (*TCL1A*) and *LGALS9* but low expression levels of *CD1*9, *CCR7* and *CXCR5*. The threshold for B-cell receptor signalling pathways is mediated by *CD19*^20^. *CCR7* and *CXCR5* direct circulating B cells into secondary lymphoid tissues, where they can be activated by antigens. In contrast, the expression of genes involved in B-cell activation (*CD83*, *Jun*, *JunB*, *Fos*, *FosB*, *ICOSLG*, *STAT3*, *CD44*, *NR4A1*) and B-cell proliferation (*EGR*-1) was abolished or dramatically downregulated, suggesting that B cells from the DC patients have defects in activation, homing, or maturation (Fig. 4d).

### DKC1_R449G iPSCs exhibit a shorter telomere length, decreased levels of hTR, reduced telomerase activity, and mislocalized DKC1

A missense variant (c.1345C > G) of DKC1 resulted in the substitution of a highly conserved amino acid, p.Arg449Gly, which is localized in the conserved nuclear localization signal domain of DKC1 (Supplementary Fig. 2). DKC1 is required for the stability and maturation of hTR^21–23^. To study the effects of the *DKC1* alterations on telomere maintenance, iPSCs were generated from the PBMCs of the younger patient and his father. We obtained one clone (WT_iPSC_F) of iPSCs from the patients’ father and two independent clones (R449G_iPSC_1 and 2) of iPSCs from the younger patient. Compared to iPSCs derived from a healthy donor (WT_iPSCs), all the iPSCs we reprogrammed showed all the hallmarks of pluripotency, including characteristic morphology (Supplementary Fig. 3a) and gene expression (Supplementary Fig. 3b). Sanger sequencing analysis showed that both R449G_iPSC_1 and R449G_iPSC_2 but not WT_iPSC and WT_iPSC_F contained the c.1345C > G variant in *DKC1* (Supplementary Fig. 3c). Karyotype analysis of WT_iPSC, WT_iPSC_F, and R449G_iPSC_1 was normal and revealed 46 chromosomes with X and Y. However, R449G_iPSC_2 had an abnormal chromosome karyotype at r(18) (p11.31q21.1) (Supplementary Fig. 3d).

**Fig. 5:**
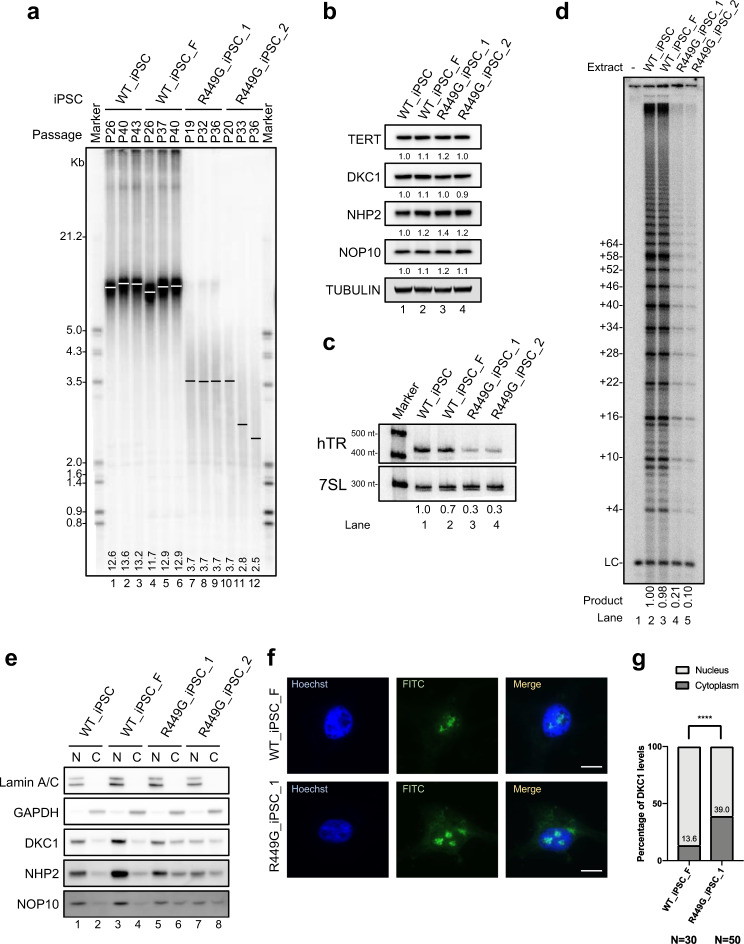
DKC1_R449G iPSCs exhibit a shorter telomere length, decreased levels of hTR, reduced telomerase activity, and mislocalized DKC1. **a** Telomere lengths determined by TRF analysis of gDNA prepared from WT_iPSC, WT_iPSC_F, R449G_iPSC_1 and R449G_iPSC_2 cells. **b** Western blot analysis of cell lysates from WT_iPSC, WT_iPSC_F, R449G_iPSC_1 and R449G_iPSC_2 cells. TUBULIN served as a loading control. **c** Northern blot analysis of hTR from WT_iPSC, WT_iPSC_F, R449G_iPSC_1 and R449G_iPSC_2 cells. A probe against 7SL RNA served as a loading control. **d** Direct telomerase activity assay of cell lysates from WT_iPSC, WT_iPSC_F, R449G_iPSC_1 and R449G_iPSC_2 cells. 18-nucleotide telomeric primers (TTAGGG)3 were used. A ^32^P end-labeled 18-nucleotide telomeric primers (TTAGGG)3 was added as loading control (LC). The numbers on the left (+4, +10, +16, +22, +28, etc.) indicate the number of nucleotides added to the telomeric-primer for each major band seen. **e** Western blot analysis of nuclear and cytosolic fractions from WT_iPSC, WT_iPSC_F, R449G_iPSC_1 and R449G_iPSC_2 cells. Lamin A/C served as a nuclear marker, and GAPDH served as a cytosolic marker. **f** Immunofluorescence of DKC1 in WT_iPSC_F and R449G_iPSC_1 cells. The scale bar represents 10 µm. **g** Bar graph illustrating the distribution of DKC1 in the cytosolic and nuclear fractions.

We examined the effect of the R449G mutation on telomere maintenance. The telomere lengths of R449G_iPSC_1 and R449G_iPSC_2 (Fig. 5a, lanes 7~12) were shorter than those of WT_iPSC and WT_iPSC_F (Fig. 5a). Although DKC1_R449G mutant iPSCs did not show significant reductions in the levels of key protein components of telomerase, including TERT, DKC1, NHP2, and NOP10 (Fig. 5b), however, they showed a dramatic 70% reduction in the steady-state level of hTR (Fig. 5c) and a decrease in telomerase activity (Fig. 5d). Cell fractionation of iPSCs revealed that the cytoplasmic proportions of DKC1 and other core H/ACA components (NHP2 and NOP10) increased in both R449G_iPSC_1 and R449G_iPSC_2 compared to WT_iPSC and WT_iPSC_F (Fig. 5e). The localization of DKC1_R449G was further confirmed by an immunofluorescence assay (Fig. 5f, g). Compared to WT_iPSC_F, the cytoplasmic proportion of DKC1_R449G was increased by more than 3-fold in R449G_iPSC_1 (Fig. 5e, f). These data suggest that the DKC1_R449G variant causes mislocalization of DKC1 to the cytoplasm, leading to reduced levels of hTR and telomere shortening.

### Inhibition of oligoadenylation of hTR restores telomerase activity and telomere length in DKC1 mutant iPSCs

**Fig. 6:**
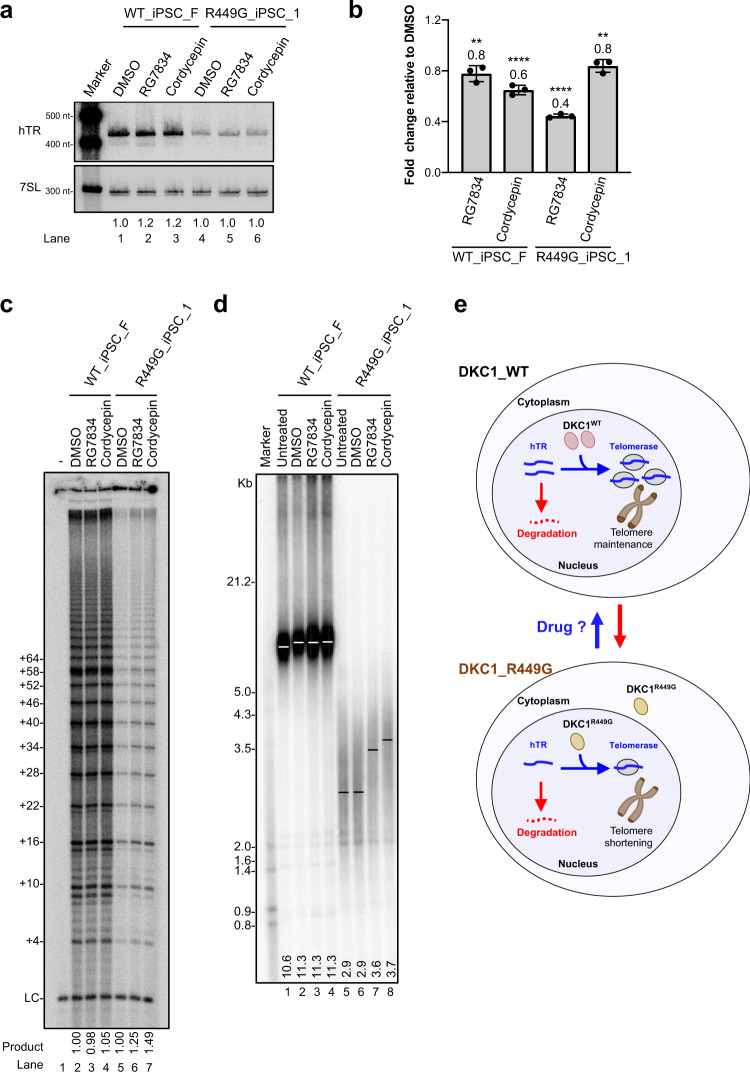
Inhibition of oligoadenylation of hTR restores telomerase activity and telomere length in DKC1 mutant iPSCs. **a** Northern blot analysis of hTR from WT_iPSC_F, R449G_iPSC_1 and R449G_iPSC_2 cells treated with RG7834, Cordycepin, or DMSO. A probe against 7SL RNA served as a loading control. **b** Total RNA prepared from WT_iPSC_F and R449G_iPSC_1 cells treated with RG7834, Cordycepin, or DMSO was subjected to qRT–PCR for oligoadenylated hTR. Bar graph of the mean fold change relative to DMSO-treated samples and normalized to GAPDH, ATP5b, and HPRT. Mean values were calculated from triplicate qRT–PCR experiments with three biological replicates, with bars representing standard error of the mean (s.e.m.). **c** Direct telomerase activity assay of cell lysates from WT_iPSC_F and R449G_iPSC_1 cells treated with RG7834 or cordycepin. 18-nucleotide telomeric primers (TTAGGG)3 were used. A ^32^P end-labeled 18-nucleotide telomeric primers (TTAGGG)3 was added as loading control (LC). The numbers on the left (+4, +10, +16, +22, +28, etc.) indicate the number of nucleotides added to the telomeric-primer for each major band seen. **d** Telomere lengths determined by TRF analysis of gDNA prepared from WT_iPSC_F and R449G_iPSC_1 treated with RG7834, Cordycepin, or DMSO. **e** Schematic illustrating the dysfunction of DKC1_R449G in telomere maintenance.

Inhibition of PAPD5-mediated oligoadenylation has been suggested to be a means of lengthening telomere length^21,24–27^. Therefore, we treated R449G_iPS cells with RG7834, a PAPD5 inhibitor, or cordycepin (3′ deoxyadenosine). RG7834 was previously reported to reverse the molecular cause of DC, increase hTR levels, and lengthen telomeres in iPSCs carrying DKC1 mutations^27,28^. Cordycepin could induce chain termination due to the absence of a 3′ hydroxyl moiety and therefore terminate poly (A) tail formation^29^. Analysis of hTR purified from RG7834- or cordycepin-treated WT_iPSC_F and R449G_iPSC_1 demonstrated minor effects on the steady-state levels of hTR (Fig. 6a) but caused a reduction in the fraction of oligoadenylated hTR (Fig. 6b), suggesting that PAPD5-mediated oligoadenylation was attenuated. In addition, the increased levels of telomerase were observed (Fig. 6c). Remarkably, both RG7834 and cordycepin partially restored telomere length in R449G_iPSC_1 (Fig. 6d, lanes 7 and 8). These data suggest that the inhibition of oligoadenylation mediated by PAPD5 is a strategy to attenuate telomere shortening caused by telomerase dysfunction.

## Discussion

In summary, our data indicate that the NLS domain of DKC1 is important for telomerase accumulation and telomere maintenance. A missense variant (R449G) causes Hoyeraal-Hreidarsson syndrome. Biogenesis of human telomerase and degradation of hTR undergo kinetic competition, which determines telomere length homeostasis (Fig. 6e). The DKC1_R449G variant results in the cytoplasmic accumulation of DKC1, causing reduced levels of hTR (Fig. 5c), reduced telomerase activity (Fig. 5d), and telomere shortening (Fig. 5a). PAPD5 (TRF4-2) is a non-canonical poly(A) polymerase and is responsible for oligoadenylation of hTR with MTR4 playing a role downstream of oligoadenylation in the degradation of hTR by the RNA exosome, which is in competition with PARN-mediated hTR maturation^24^. In DKC1- or PARN-deficient cells, hTR is not only destabilized, but also accumulates in cytoplasmic puncta called cyTER bodies^21^. Inhibition of oligoadenylation by a PAPD5 inhibitors increases hTR levels, reduces the fractions of 3′-end oligoadenylation of hTR, and elongates telomeres^27,28^. In addition, subcellular localization of hTR is corrected upon RG7834 treatment. Supporting the role of PARN in competing PAPD5-mediated hTR degradation by the RNA exosome, more obvious effect of PAPD5 inhibitors on hTR is observed in PARN-deficient cells than DKC1-dificient cells^21,27,28^. Although our data indicate that treatment of R449G_iPSC_1 with RG7834 or cordycepin reduced 3′ end oligoadenylation of hTR (Fig. 6b), increased telomerase activity (Fig. 6c), and elongated telomere (Fig. 6d), there was no significant increase in hTR levels (Fig. 6a). In addition, there was no correlation between the decrease in oligoadenylated hTR and the change in telomere length upon RG7834 and cordycepin treatment (Fig. 6b, d). Whether these discrepancies between our case and other cases in previous studies are the DKC1 mutant type-specific remains to be investigated. Further studies are still needed to examine the effects of these small compounds on telomere disorder syndrome and age-related degenerative disease.

We examined the transcriptomic profiles of immune cells in a DC patient carrying a missense mutation (c.1345C > G) in the conserved nuclear localization signal domain of *DKC*1 using single-cell RNA-seq. We found that the DC patient exhibited immunological features of defective NLRP3 inflammasomes in monocytes, immature NK dominance with fewer cytotoxic NK cells (CD56^dim^ NK cells), and defective B-cell activation or maturation, suggesting that the causes of poor immune responses in the DC patient are caused by not only leukopenia but also dysfunction of immune cells. The NLRP3 inflammasome plays a crucial role in modulating the caspase-1-dependent release of proinflammatory cytokines, which in turn controls the early phase of host defence, inflammation, and subsequent activation of adaptive immunity. To our knowledge, this is the first report of a clinical phenotype showing loss of function of NLRP3. Interestingly, the human NLRP3 gene resides in the subtelomeric region of chromosomes that are deficient in DNA repair mechanisms. How telomere shortening affects the expression of genes in the subtelomeric region remains remain unclear. There are several limitations of our study. Our sample size was small, and patients varied with respect to the timing of clinical presentation during the progression of disease, which could influence the transcriptional landscape. Whether NLRP3 deficiency in Hoyeraal-Hreidarsson syndrome could be a novel component of innate immunity defects remains to be investigated.

## Methods

### Exome sequencing

Whole-exome sequencing (WES) was performed as previously reported^30^. In brief, after DNA extraction, exome capture was performed with the TruSeq Exome Capture Kit (Illumina), and sequencing for the three participants (patient and both parents) was conducted using the NextSeq500 mid output system (Illumina) with a 75-bp paired-end run. The sequences were aligned to the human reference genome (GRCh37), and variant calling was performed using the Genome Analysis Toolkit (GATK V3.5, Broad Institute)^31^. Variants were first annotated by Variant Studio (V3.0, Illumina) and wANNOVAR (http://wannovar.wglab.org/)^32^. Candidate variants were checked with ClinVar (https://www.ncbi.nlm.nih.gov/clinvar/). The pathogenicity of variants was classified according to the ACMG guidelines^33^.

### Karyotype analysis

Karyotype analysis of iPSCs was conducted as follows: iPSCs that reached eighty percent confluence in 35 mm dishes were harvested after adding 10 µL of colcemid for 15 hours. Then, 2 ml 0.28% KCl was added twice for 50 minutes. Then, 2 mL methanol/acetic acid (3/1) was added for fixation. G banding was stained using 0.25% trypsin followed by Wright’s solution (pH 7.0 Gurr buffer/Wright’s = 3/1).

### iPSC derivation, culture, and drug treatment

PBMCs from whole blood were separated by Ficoll-Hypaque density gradients and stored in liquid nitrogen. iPSCs were derived from the PBMCs of the patient and the patient’s father. PBMCs were reprogrammed using Sendai virus according to the manufacturer’s instructions. For feeder-free culture, iPSCs were maintained in StemFlex completed medium containing StemFlex^TM^ Basal Medium (Gibco, Cat. No. A33493-01) and StemFlex^TM^ Supplement (10X) (Gibco, Cat. No. A33492-01) on Matrigel hESC-qualified Matrix (Corning, Cat. No. 354277) at 37 °C in a humidified atmosphere containing 5% CO_2_. To prepare Matrigel-coated dishes, Matrigel hESC-qualified matrix supplemented with 25 mL of DMEM/F-12, HEPES (Gibco, Cat. No. 11330032) was added to 6-well plates (1 mL/well) or 100 mm dishes (7 mL/dish) and incubated at 37 °C for 2 hours. iPSCs were subcultured when the cells were in log phase, and the cells were detached by using Accutase^®^ (Innovative Cell Technologies, Inc., Cat. No. #AT-104) and incubated in an incubator at 37 °C and 5% CO_2_ for 5 min. The detached cells were centrifuged at 1000 rpm (Kubota 2010 tabletop centrifuge) for 5 min. Before seeding the cells, Matrigel matrix supplemented with DMEM/F-12 was aspirated from the culture dish. iPSCs were seeded on a Matrigel-coated dish containing StemFlex complete medium and 10 μM Y-27632 dihydrochloride (Rock inhibitor) (Sigma–Aldrich, Cat: Y0503-5MG) at 37 °C with 5% CO_2_, and then the medium was changed every day. For RG7834 and cordycepin treatment, iPSCs were maintained in StemFlex complete medium with 1 µM RG7834 (MedChemExpress (MEC), Cat. No.: HY-117650A), 1 µM Cordycepin (MedChemExpress (MEC), Cat. No.: HY-N0262) or dimethyl sulfoxide (Sigma–Aldrich, Cat: D4540-100ML) as a control on Matrigel hESC-qualified matrix. The cells were subcultured as mentioned before.

### DNA sequencing and genetic analysis

DNA sequencing of gDNA from PBMCs and iPSCs from healthy donors and DC patients was performed by polymerase chain reaction (PCR) amplification of the specific region of exon 14 for *DKC1*. The primers used for PCR and Sanger sequencing are listed in Supplementary Table 2. Each PCR (100 µl) contained 100 ng of DNA, 1X KAPA HiFi Fidelity Buffer, 0.3 mM KAPA dNTP, 0.3 µM each primer, and 0.005 U/μl KAPA HiFi DNA Polymerase (Kapa Biosystems, Cat: KK2102). The reactions were carried out in a T100^TM^ Thermal Cycler (Bio Rad) under the following conditions: one cycle at 95 °C for 3 min, followed by 30 cycles of 20 s at 98 °C, 15 s at 50 °C and 30 s at 72 °C, with a final cycle at 72 °C for 1 min. PCR products were purified using a PCR extraction kit (TOOLS, Cat: TT-B14-3) according to the manufacturer’s instructions.

### Genomic DNA extraction and qPCR

Genomic DNA was prepared from iPSC pellets (5 × 10^6^ cells) with GenElute^TM^ Mammalian Genomic DNA Miniprep Kits (Sigma–Aldrich, Cat. No: G1N350-1KT) according to the manufacturer’s instructions. To evaluate telomere lengths, polymerase chain reaction (PCR)-based telomere length analysis methods have been developed. In this study, telomere length was quantified by comparing the amount of the telomere amplification product (T) to that of a single-copy gene (S, 36B4). The T/S ratio was then calculated to yield a value that correlated with the average telomere length. Each reaction contained 5 ng/μl genomic DNA or standard templates mixed with iQ™ SYBR® Green Supermix (Bio–Rad, Cat. No. 1708882) and primers. qPCR was performed in a CFX96^TM^ Real-Time System, C1000 Touch^TM^ Thermal Cycler (Bio–Rad), with the following cycling conditions: initial denaturation at 95 °C for 10 min and 40 cycles at 95 °C for 15 sec and 60 °C for 1 min. Graphing and statistical analysis of qPCR results were performed using Prism 8 (GraphPad). Standard telomere oligonucleotides, standard single-copy gene (36B4) nucleotides, and primers used for qPCR are listed in Supplementary Table 3.

### Terminal restriction fragment (TRF) analysis

Genomic DNA (2.2 µg) from iPSCs was digested with *Hin*f I (New England Biolabs, Cat. No: #R0155S) and *Rsa* I (New England Biolabs, Cat. No: #R0167L) restriction enzyme in 10X CutSmart® Buffer (NEB, Cat. No: #B7204S) at 37 °C overnight. The digested gDNA fragments were separated on a 1% SeaKem® LE agarose gel (Lonza, Cat. No: 50002) by electrophoresis at 120 V for 12 h, followed by capillary transfer to a Hybond^TM^-N^+^ nylon transfer membrane (GE Healthcare, Cat. No: RPN303B) in 10X SSC for 14.5 h. DNA was subsequently crosslinked to the membrane twice at 120 mJ in a UV Stratalinker 1800 (Stratagene, 254 nm, 120 mJ). The blot was prehybridized in Church buffer at 65 °C for an hour and then hybridized with ^32^P-α-dCTP-labelled (TTAGGG)_3_ overnight. The blot was exposed to a phosphor image screen (Fujifilm) at room temperature overnight. Phosphor images were obtained with an Amersham Typhoon 5 scanner (Cytiva). The telomere length images were quantified and analysed by ImageQuantTL software (Cytiva). All blots derive from the same experiment and were processed in parallel.

### Single-cell RNA sequence capture, library construction, and sequencing

Single-cell capture and downstream library construction were performed using Chromium Next GEM Single Cell 3′ Reagent Kits v 3.1 (10x Genomics; PN-1000121, PN-1000127, and PN-1000213) for PBMCs from the DC patient and age-matched controls, according to the manufacturer’s protocol. Briefly, a total of approximately 8600 single cells, 50 µl of barcoded gel beads and 45 µl partitioning oil were loaded into Chromium Next GEM Chip G to generate nanoliter-scale gel beads-in-emulsion (GEMs). Afterwards, the polyadenylated mRNAs were reverse transcribed inside each GEM, and the full-length cell-barcoded cDNA was amplified via PCR to generate sequencing libraries. Library quality was assessed by using the Qubit 4.0 Fluorometer (Thermo Scientific) and a Qsep100^TM^ system (Bioptic, Taiwan) to determine the library concentration and library size, respectively. In general, fragments of approximately 450–500 bp in size were expected for single-cell 3ʹ gene expression libraries. The effective concentrations of the library were assessed by Q-PCR. The qualified libraries were pooled according to the effective concentration and expected data volume. The library was sequenced on Illumina NovaSeq 6000 sequencers according to read length: 28 bp Read 1 (16 bp single-cell barcode, 10x barcode; 12 bp unique molecular identifier, UMI), 91 bp Read 2 (transcript insert or feature barcode in the case of the cell hashing library), and 8 bp i7 Index (sample index). TruSeq Read 1 and TruSeq Read 2 are standard Illumina sequencing primer sites used in the paired-end sequencing of single-cell 3ʹ gene expression libraries.

### Single-cell gene expression analysis

In the Chromium Single-Cell Software Suite, Cell Ranger (*cellranger count*) was used to perform sample demultiplexing and generate feature-barcode matrices. Sequences were mapped onto the human reference genome (GRCh38) provided by 10X Genomics. Multiple samples were aggregated by “*cellranger aggr*” without depth normalization. Unique molecular identifier (UMI) count matrices were imported into R (v3.6.0) and processed with the R package Seurat (v3.2.1)^34,35^. Log-normalized expression values were obtained by the “*NormalizeData*” function of the Seurat package. Specifically, “*LogNormalize*” was set by default in this function, and the gene counts for each cell were divided by the total counts and multiplied by the *scale.factor* (default = 10,000) and then natural-log transformed. Further analysis, including quality control, the identification of highly variable genes (HVGs), dimensionality reduction, and standard unsupervised clustering algorithms, was performed using the Seurat package. Cells were filtered out before downstream analysis if (1) the percentage of mitochondrial genes was >20% or (2) the number of genes was <200 or >Q3 + 1.5 IQR of the population. HVGs, which are often used to keep the most informative variations in the scRNA-seq data^36,37^, were set in the “*FindVariableFeatures*” function of the Seurat package; the number of HVGs was defined by the median gene number in the population, while “*nfeatures* = *2000*” was defined if the median was below 2000. Visualizing a high-dimensional single-cell dataset is critical for interpretation of the results. Cell clustering was performed by using dimensional reduction techniques and t-distributed stochastic neighbour embedding (tSNE)^38,39^. Note that in Seurat, both tSNE and UMAP were performed after PCA (“*RunPCA*” function). The “*RunTSNE*” and “*RunUMAP*” functions in Seurat were set with “*dims* = *1:20*”. To evaluate the batch effect, batch mixing was used to quantify the extent of intermingling of cells from different batches^40,41^. If necessary, the mutual nearest neighbour (MNN)^40^ method was used for batch effect correction with the “*fastMNN*” function of SeuratWrappers (v0.3.0). To identify the cell types captured by scRNA-seq in an unbiased fashion, an automatic annotation method, SingleR (v1.0.1), was performed^7^; this method correlates each cell with reference transcriptomic datasets independently. Differentially expressed gene (DEG) analysis of the two conditions was performed in Python using sSeq^42^ (total UMI count <900) and edgeR^43,44^ (total UMI count >900), which is based on negative binomial distribution and asymptotic beta testing, respectively^42–44^. We followed the method as stated in Cell Ranger and the Loupe Cell Browser, and the source code is available in the 10X Genomics GitHub repository (https://github.com/10XGenomics/cellranger).

### RNA extraction and reverse transcription PCR

Total RNA was isolated from the iPSC pellets (1 × 10^7^ cells) using Ambion TRIzol^®^ Reagent (Life Technologies, Cat. No: 15596018) according to the manufacturer’s instructions, followed by DNase I (New England Biolabs, Cat: M0303 L) treatment at 37 °C for 60 min. For cDNA synthesis, the reverse transcription reaction was performed according to the manufacturer’s instructions of the SuperScript^TM^ IV First-Strand Synthesis System (Thermo Fisher Scientific, Cat. No. 18091050). In brief, 2 µg of total RNA was primed with 50 µM Oligo d(T)_20_ primer or 50 ng random hexamers in 10 mM dNTP mix and DEPC-treated water. PCRs were carried out in a T100^TM^ Thermal Cycler (Bio-Rad) under the following conditions: one cycle at 65 °C for 5 min and 4 °C for 1 min. Then, 1 µl of SuperScript^TM^ IV RT was added to the reaction mixture containing ribonuclease inhibitor (TOOLS, Cat: TTG-RI01), 5 mM DTT, and 1X SSIV Buffer, followed by incubation at 50 °C for 1 h, 80 °C for 10 min, 37 °C for 1 min and 4 °C for 1 min. Finally, 1 µl of RNase H (2 U/µl) was added to the samples to remove RNA at 37 °C for 20 min, 65 °C for 10 min, and 4 °C for 10 min. RT–PCR was performed with primers targeting pluripotent stem cell markers (OCT4, SOX2, NANOG) using a standard Taq Reaction Buffer Pack (New England Biolabs, Cat. No. B9014S and M0273S) in a T100^TM^ Thermal Cycler (Bio-Rad) under the following conditions: one cycle at 95 °C for 2.5 min, followed by 35 cycles of 30 s at 95 °C, 30 s at 55 °C and 1 min at 68 °C, with a final cycle at 68 °C for 5 min. The primers used for RT–PCR are listed in Supplementary Table 4.

### qRT–PCR

Quantitative reverse transcription-polymerase chain reaction (qRT–PCR) was performed with the SYBR Green method. The 25-fold diluted oligo dT or random hexamer priming cDNA was amplified with the primers shown in Supplementary Table 5 and was performed with the CFX384^TM^ Real-Time PCR System, in a C1000 Touch^TM^ Thermal Cycler (Bio-Rad) using iQ™ SYBR® Green Supermix (Bio-Rad, Cat. No. 1708882). The results were normalized to the GAPDH, ATP5β, and HPRT reference genes and measured by CFX Maestro software (Bio-Rad). Graphing and statistical analysis of qRT–PCR results were performed using Prism 8 (GraphPad).

### Northern blotting

Total RNA (10 µg) was separated on a 4% polyacrylamide (29:1) gel containing 8 M urea at 20 W for 1 h and then transferred to a Hybond-N^+^ nylon transfer membrane (GE Healthcare, Cat. No: RPN303B) at 400 mA for 1 h in 0.5X TBE buffer. RNA was cross-linked to the membrane in a Stratalinker (Stratagene, 120 mJ). The blot was prehybridized in Church buffer at 65 °C (for hTR) or at 42 °C (for oligonucleotide probe) for an hour. Hybridizations with radiolabelled probes were performed in Church buffer at 65 °C (for hTR, probes were generated by nick translation of a polymerase chain reaction (PCR) fragment with ^32^P-α-dCTP) and 42 °C (for oligonucleotide probe against 7SL, which was labelled with ^32^P-γ-ATP by T4 PNK kinase). The oligonucleotide sequences are listed in Supplementary Table 6. All blots derive from the same experiment and were processed in parallel.

### Cell lysis and Western blotting

All iPSC pellets were lysed in lysis buffer containing 0.5% CHAPS, 50 mM Tris-HCl (pH 8.0), 50 mM KCl, 1 mM MgCl_2_, 1 mM EGTA, 10% glycerol, 5 mM DTT, and 1 mM PMSF. Total lysates were incubated at 4 °C for 1 h on a rotator, and insoluble material was removed by centrifugation at 21,130 × *g* at 4 °C for 10 min. Protein concentration was measured using the protein assay dye (Bio-Rad, Cat. No. 5000006). Twenty micrograms of protein were resolved on 4–20% Bis-Tris gradient gels (GenScript, Cat. No: M00657) at 180 V for 40 min and then transferred to polyvinylidene fluoride (PVDF) membranes (BIO-RAD Immun-Blot®, Cat. #1620177) at 100 V for 1 h. Five percent skim milk in washing buffer was used as a blocking reagent. The prestained protein ladder (Omics Bio, Cat: 02101-250) was used as a marker; ɑ-tubulin (1:5000, ABclonal, Cat. No: AC012) was used as a loading control. Cytiva software was used. The antibodies used in this study are listed in Supplementary Table 7. All blots derive from the same experiment and were processed in parallel.

### Cytoplasmic/nuclear fractionation

iPSCs (2 × 10^6^) were washed with DPBS (Biological Industries, Cat. No: 02-023-1A) and lysed in 50 µl of fresh buffer A (0.05% Triton X-100, 10 mM HEPES-KOH, pH 7.9, 10 mM KCl, 1.5 mM MgCl_2_, 10% glycerol, 0.34 M sucrose, 1 mM DTT, supplemented with protease inhibitor cocktail) at 4 °C for 10 min. The cytosolic fraction was collected and clarified at 500 × g at 4 °C for 5 min twice. The cell monolayer was then washed three times with DPBS and resuspended in a new buffer A. Both nuclear and cytoplasmic fractions were analysed by Western blotting as mentioned before. The antibodies used in this study are listed in Supplementary Table 7.

### Immunofluorescence assay

The iPSCs were fixed with 4% paraformaldehyde at RT for 20 min, permeabilized with 0.1% Triton X-100 at RT for 5 min, and blocked with 1% BSA in DPBS at RT for an hour, followed by incubation with the primary antibody against dyskerin (H-3) (Santa Cruz Biotechnology, Cat. No. sc-373956, 1:500 dilution) at 4 °C overnight. The slide was washed three times with DPBS and then incubated with fluorescein (FITC)-conjugated AffiniPure goat anti-mouse IgG (H + L) (Jackson ImmunoResearch, Cat. No. 115-095-003, 1:100 dilution) secondary antibody at 37 °C for 1 h. Nuclei were stained with bisbenzimide H 33258 (Sigma–Aldrich, Cat. No. B2883-1 g, 1:1000 dilution) for 10 min at 4 °C after washing the cells with DPBS. After the indicated treatments, coverslips were mounted on glass slides with Fluoromount^TM^ Aqueous Mounting Medium (Sigma–Aldrich, Cat. No. F4680-25ML) and photographed under an Axio Imager 2 fluorescence microscope (ZEISS). Acquired images were quantified by using ImageJ/Fiji software. Graphing and statistical analysis of the immunofluorescence assay results were performed in Prism 8 (GraphPad). The antibodies used in this study are listed in Supplementary Table 8.

### Telomerase activity assay

Telomerase activity reactions were performed as 10 µl reactions with: 50 mM Tris-HCl pH 8.0, 50 mM KCl, 1 mM MgCl_2_, 1 mM spermidine, 5 mM DTT, 1 mM dATP, 1 mM dTTP, 10 µM dGTP, and 0.75 µM ^32^P-dGTP (3000 Ci/mmol), 1 µM telomeric primer (TTAGGG)3, and 2 µg cell extract at 37 ^o^C for 2 hours. Reactions were stopped with 10 µl of 1 mg/ml proteinase K. DNA was extracted with phenol/chloroform equilibrated with 50 mM NaOAc (pH 7.0) and ethanol precipitated with 2.5 M ammonium acetate and 10 µg of glycogen at −80 ^o^C overnight. Reactions were then centrifuged for 20 min at 14,000 r.p.m., and the pellets were washed with 1 ml of 70% ethanol. The dried pellets were then resuspended in 5 µl of 80% formamide loading buffer. Reaction products were analysed on a 10% polyacrylamide (19:1) gel containing 8 M urea. All blots derive from the same experiment and were processed in parallel.

### Reporting summary

Further information on research design is available in the Nature Research Reporting Summary linked to this article.

## Supplementary information

Supplementary information

Reporting Summary

Raw data of qPCR related to Figure 1b

Raw data of qPCR related to Figure 1c

Raw data of RT-qPCR related to Figure 6b

## Data Availability

The data that support the findings of this study are available from the corresponding authors on reasonable request. qPCR, RT-qPCR, and DNA sequencing data are available in the supplementary information. Whole exome sequencing and single-cell RNA-Seq datasets that support the findings of this study have been deposited to NCBI SRA database with the BioProject ID: PRJNA880366.

## Code availability

The original code for Seurat is available at https://github.com/satijalab/seurat. Code for scRNA processing pipeline is available at https://github.com/phoebee-h/MS_GenomeMedicine.

## References

[1] Heiss NS (1998). X-linked dyskeratosis congenita is caused by mutations in a highly conserved gene with putative nucleolar functions. Nat. Genet..

[2] AlSabbagh MM (2020). Dyskeratosis congenita: a literature review. J. Dtsch Dermatol. Ges..

[3] Aalfs CM, van den Berg H, Barth PG, Hennekam RC (1995). The Hoyeraal-Hreidarsson syndrome: the fourth case of a separate entity with prenatal growth retardation, progressive pancytopenia and cerebellar hypoplasia. Eur. J. Pediatr..

[4] Hreidarsson S, Kristjansson K, Johannesson G, Johannsson JH (1988). A syndrome of progressive pancytopenia with microcephaly, cerebellar hypoplasia and growth failure. Acta Paediatr. Scand..

[5] Savage, S.A. Beginning at the ends: telomeres and human disease. *F1000Res* **7**(2018). 10.12688/f1000research.14068.1 PMC5931273 29770205

[6] Niewisch MR (2022). Disease progression and clinical outcomes in telomere biology disorders. Blood.

[7] Aran D (2019). Reference-based analysis of lung single-cell sequencing reveals a transitional profibrotic macrophage. Nat. Immunol..

[8] Moar P, Tandon R (2021). Galectin-9 as a biomarker of disease severity. Cell Immunol..

[9] Jorgovanovic D, Song M, Wang L, Zhang Y (2020). Roles of IFN-gamma in tumor progression and regression: a review. Biomark. Res.

[10] Hayden MS, Ghosh S (2011). NF-kappaB in immunobiology. Cell Res..

[11] Patel AA (2017). The fate and lifespan of human monocyte subsets in steady state and systemic inflammation. J. Exp. Med..

[12] Ong SM (2018). The pro-inflammatory phenotype of the human non-classical monocyte subset is attributed to senescence. Cell Death Dis..

[13] Metcalf TU (2017). Human monocyte subsets are transcriptionally and functionally altered in aging in response to pattern recognition receptor agonists. J. Immunol..

[14] Anbazhagan K, Duroux-Richard I, Jorgensen C, Apparailly F (2014). Transcriptomic network support distinct roles of classical and non-classical monocytes in human. Int Rev. Immunol..

[15] Swanson KV, Deng M, Ting JP (2019). The NLRP3 inflammasome: molecular activation and regulation to therapeutics. Nat. Rev. Immunol..

[16] Mao L (2018). Loss-of-function CARD8 mutation causes NLRP3 inflammasome activation and Crohn’s disease. J. Clin. Invest..

[17] Rex DAB (2020). A comprehensive pathway map of IL-18-mediated signalling. J. Cell Commun. Signal.

[18] Wendt K (2006). Gene and protein characteristics reflect functional diversity of CD56dim and CD56bright NK cells. J. Leukoc. Biol..

[19] Golden-Mason L (2013). Galectin-9 functionally impairs natural killer cells in humans and mice. J. Virol..

[20] Carter RH, Fearon DT (1992). CD19: lowering the threshold for antigen receptor stimulation of B lymphocytes. Science.

[21] Shukla S, Schmidt JC, Goldfarb KC, Cech TR, Parker R (2016). Inhibition of telomerase RNA decay rescues telomerase deficiency caused by dyskerin or PARN defects. Nat. Struct. Mol. Biol..

[22] Tseng CK, Wang HF, Schroeder MR, Baumann P (2018). The H/ACA complex disrupts triplex in hTR precursor to permit processing by RRP6 and PARN. Nat. Commun..

[23] Wong JM, Kyasa MJ, Hutchins L, Collins K (2004). Telomerase RNA deficiency in peripheral blood mononuclear cells in X-linked dyskeratosis congenita. Hum. Genet.

[24] Tseng CK (2015). Human Telomerase RNA processing and quality control. Cell Rep..

[25] Boyraz B (2016). Posttranscriptional manipulation of TERC reverses molecular hallmarks of telomere disease. J. Clin. Invest.

[26] Fok WC (2019). Posttranscriptional modulation of TERC by PAPD5 inhibition rescues hematopoietic development in dyskeratosis congenita. Blood.

[27] Nagpal N (2020). Small-Molecule PAPD5 Inhibitors restore telomerase activity in patient stem cells. Cell Stem Cell.

[28] Shukla S, Jeong HC, Sturgeon CM, Parker R, Batista LFZ (2020). Chemical inhibition of PAPD5/7 rescues telomerase function and hematopoiesis in dyskeratosis congenita. Blood Adv..

[29] Kondrashov A (2012). Inhibition of polyadenylation reduces inflammatory gene induction. RNA.

[30] Wu ET (2019). Critical trio exome benefits in-time decision-making for pediatric patients with severe illnesses. Pediatr. Crit. Care Med.

[31] McKenna A (2010). The Genome Analysis Toolkit: a MapReduce framework for analyzing next-generation DNA sequencing data. Genome Res..

[32] Wang K, Li M, Hakonarson H (2010). ANNOVAR: functional annotation of genetic variants from high-throughput sequencing data. Nucleic Acids Res.

[33] Richards S (2015). Standards and guidelines for the interpretation of sequence variants: a joint consensus recommendation of the American College of Medical Genetics and Genomics and the Association for Molecular Pathology. Genet. Med..

[34] Butler A, Hoffman P, Smibert P, Papalexi E, Satija R (2018). Integrating single-cell transcriptomic data across different conditions, technologies, and species. Nat. Biotechnol..

[35] Stuart T (2019). Comprehensive integration of single-cell data. Cell.

[36] Brennecke P (2013). Accounting for technical noise in single-cell RNA-seq experiments. Nat. Methods.

[37] Luecken MD, Theis FJ (2019). Current best practices in single-cell RNA-seq analysis: a tutorial. Mol. Syst. Biol..

[38] Kobak D, Berens P (2019). The art of using t-SNE for single-cell transcriptomics. Nat. Commun..

[39] Van der Maaten LJP, Hinton GE (2008). Visualizing high-dimensional data using t-SNE. J. Mach. Learn. Res..

[40] Haghverdi L, Lun ATL, Morgan MD, Marioni JC (2018). Batch effects in single-cell RNA-sequencing data are corrected by matching mutual nearest neighbors. Nat. Biotechnol..

[41] Kinchen J (2018). Structural remodeling of the human colonic mesenchyme in inflammatory bowel disease. Cell.

[42] Yu D, Huber W, Vitek O (2013). Shrinkage estimation of dispersion in Negative Binomial models for RNA-seq experiments with small sample size. Bioinformatics.

[43] Robinson MD, McCarthy DJ, Smyth GK (2010). edgeR: a Bioconductor package for differential expression analysis of digital gene expression data. Bioinformatics.

[44] Robinson MD, Smyth GK (2008). Small-sample estimation of negative binomial dispersion, with applications to SAGE data. Biostatistics.

