# Supplementary material for: A missense variant in the nuclear localization signal of DKC1 causes Hoyeraal-Hreidarsson syndrome
Source: NPJ Genom Med. 2022 Oct 30;7:64. doi: 10.1038/s41525-022-00335-8 (PMC9617742; doi:10.1038/s41525-022-00335-8)
Supplement: Supplementary file 1 — Supplementary information [file 41525_2022_335_MOESM1_ESM.pdf]

Supplementary information

Supplementary Table 1. Laboratory features of two patients with HHS

	Age (yr)	Elder brother	Younger brother	Reference
WBC (/µL)	1.6	-	7240	5000-17000 (age 1-6)
	2.8	1050 L	7450	
	3	2560 L	7620	
	3.5	-	3060 L	
	5.7	-	1030 L	
Hb (g/dL)	1.6	-	13.8 L	11.5-14 (age 1-6)
	2.8	9.8 L	10.1 L	
	3	2.6 L	12.6	
	3.5	-	10.3 L	
	5.7	-	8.6 L	
PLT (k/µL)	1.6	-	102 L	150-400 (age 1-6)
	2.8	35 L	40 L	
	3	4 L	107 L	
	3.5	-	76 L	
	5.7	-	36 L	
Neutrophils (/µL)	1.6	--	4699	1000-8500 (age 1-6)
	2.8	473 L	6362	
	3	2289	5600	
	3.5	-	1888	
	5.7	-	900 L	
Lymphocytes (/µL)	1.6	-	1499 L	2180-8270 (age 1-2)
	2.8	74 L	693 L	2400-5810 (age 2-5)
	3	90 L	1227 L	
	3.5	-	930 L	
	5.7	-	50 L	
Monocytes (/µL)	1.6	-	557	200-1000 (age 1-6)
	2.8	158 L	261	
	3	161 L	617	
	3.5	-	211	
	5.7	-	70 L	
$CD3^+$ T cells (/µL)	2.8	64.8 L	857.5 L	1610-4230 (age 2-5)
	3.5	-	865.1 L	
$CD4^+$ T cells (/µL)	2.8	46.8 L	551.2 L	900-2860 (age 2-5)
	3.5	-	567.5 L	
Naïve $CD4^+CD45RA^+$ T cells (/µL)	2.8	43.2 L	408.3	300-2300 (age 2-5)
	3.5		460.5	
$CD8^+$ T cells (/µL)	2.8	16.5 L	316.4 L	630-1910 (age 2-5)

	3.5			279.1	L	
$CD19^+$ B cells (/μL)	2.8	0.5	L	122.5	L	700-1300 (age 2-5)
	3.5	-		46.5	L	
$CD56^+$ NK cells (/μL)	2.8	6.0	L	20.4	L	61-510 (age 2-5)
	3.5	-		18.6	L	
IgG (mg/dL)	2.5	337.0	L	-		419-1274 (age 2-3)
	2.8	673		774		
	3.5	-		1150		569-1597 (age 3-5)
	5.3	-		1050		
IgA (mg/dL)	2.8	110		33.4		19-235 (age 2-3)
	3.5	-		38.1		55-152 (age 3-5)
	5.3	-		25.9	L	
IgM (mg/dL)	2.8	37.6		133	H	28-113 (age 2-3)
	3.5	-		309	H	22-100 (age 3-5)
	5.3	-		118		

Abbreviations: high, H; low, L

Supplementary Table 2. Oligonucleotides used for sequencing the *DKC1* gene

Name	Sequence	Target
CKOligo-315	FW: TCAAGGCTTCTTGGATTTGG	DKC1
CKOligo-316	RV: AAGAGTAACAAAGCTGGTAC	

Supplementary Table 3. Oligonucleotides used for qPCR and standard templates

Name	Oligomer sequence (5’ – 3’)	Description
CKOligo-237	TTAGGGTTAGGGTTAGGGTTAGGGTTAGG GTTAGGGTTAGGGTTAGGGTTAGGGTTAG GGTTAGGGTTAGGGTTAGGGTTAGGG	Telomere Standard
CKOligo-238	CAGCAAGTGGGAAGGTGTAATCCGTCTCC ACAGACAAGGCCAGGACTCGTTTGTACC CGTTGATGATAGAATGGG	36B4 (SCG) Standard
CKOligo-239	CGGTTTGTTTGGGTTTGGGTTTGGGTTTG GGTTTGGGTT	Telomere-F

CKOligo-240	GGCTTGCCTTACCCTTACCCTTACCCTTAC CCTTACCCT	Telomere-R
CKOligo-241	CAGCAAGTGGGAAGGTGTAATCC	36B4 (SCG)-F
CKOligo-242	CCCATTCTATCATCAACGGGTACAA	36B4 (SCG)-R

Supplementary Table 4. Oligonucleotides used for RT–PCR

**Name**	**Sequence**	**Target**
OCT4	F: TGTACTCCTCGGTCCCTTTC	OCT4
	R: TCCAGGTTTTCTTTCCTAGC	
SOX2	F: GCTAGTCTCCAAGCGACGAA	SOX2
	R: GCAAGAAGCCTCTCCTTGAA	
NANOG	F: CAGTCTGGACACTGGCTGAA	NANOG
	R: CTCGCTGATTAGGCTCCAAC	

Supplementary Table 5. Oligonucleotides used for qRT–PCR

**Name**	**Sequence**	**Target**
CKOligo-14	FW: GCGAAGAGTTGGGCTCTGTCA	hTR
CKOligo-15	RV: TTCCTCTTCCTGCGGCCTGAAA	
CKOligo-18	FW: TGCTGAGGATTTGGAAAGGG	HPRT
CKOligo-19	RV: ACAGAGGGCTACAATGTGATG	
CKOligo-20	FW: GATCCTCTAGACTCCACCTCTC	ATP5β
CKOligo-21	RV: AGAAAGTTCATCCATACCCAGG	
CKOligo-22	FW: ACATCGCTCAGACACCATG	GAPDH
CKOligo-23	RV: TGTAGTTGAGGTCAATGAAGGG	

Supplementary Table 6. Oligonucleotides used for probe for Northern blotting

Name	Sequences		Target
CKOligo-1	FW	GGGTTGCGGAGGGTGGGC	hTR probe generated by PCR
CKOligo-2	RV	CCGACTTTGGAGGTGCCTTC	
CKOligo-5	GGAGGTCACCATATTGATGCCGAACTTAGT		7SL probe

Supplementary Table 7. Antibodies used for Western blotting

**Antibodies**	**Source**	**Cat. No.**	**Dilution**
**Loading control**			
α-Tubulin	ABclonal	AC012	1:5000
**Nuclear and cytosolic marker**			
Lamin A/C	ABclonal	A19524	1:50000
GAPDH	ABclonal	AC027	1:100000
**Secondary antibody**			
Goat anti-Mouse IgG- h+l DyLight® 680 conjugated	Bethyl Laboratories	A90-516D6	1:5000
Goat anti-Rabbit IgG (H+L)-HRP	CROYEZ	C04003	1:5000
**Primary antibody**			
DKC1	Bethyl Laboratories	A302-591A	1:2000
NOP10	ABclonal	A18250	1:1000
NHP2	Proteintech Group	15128-1-AP	1:500
TERT	Abcam	ab32020	1:1000

Supplementary Table 8. Antibodies used for immunofluorescence assays

**Antibodies**	**Source**	**Cat. No.**	**Dilution**
**Primary antibody**			
Dyskerin (H-3)	Santa Cruz Biotechnology	sc-373956	1:500
**Secondary antibody**			
Fluorescein (FITC)-conjugated AffiniPure Goat Anti-Mouse IgG (H+L)	Jackson ImmunoResearch	115-095-003	1:100

Supplementary Figure

Supplementary Figure 1

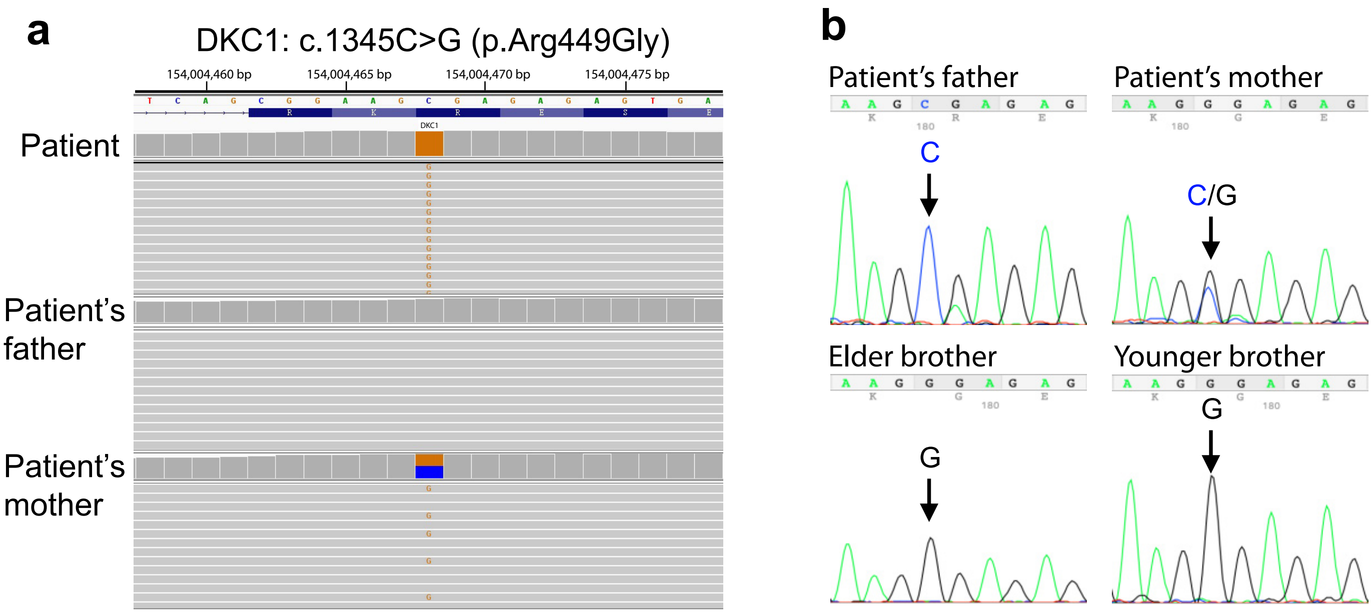

Supplementary figure 1. Genomic characterization of our patients
a. Exome sequencing showing the inheritance of the DKC1 mutation from the mother to the sibling. **b.** Sanger sequencing of DKC1 gDNA prepared from PBMCs of all members of the family. The patient's father is indicated by C (normal). The patient's mother and sibling are indicated to have a DKC1 c.1345C> G (p. R449G) mutation.

Supplementary Figure 2

a

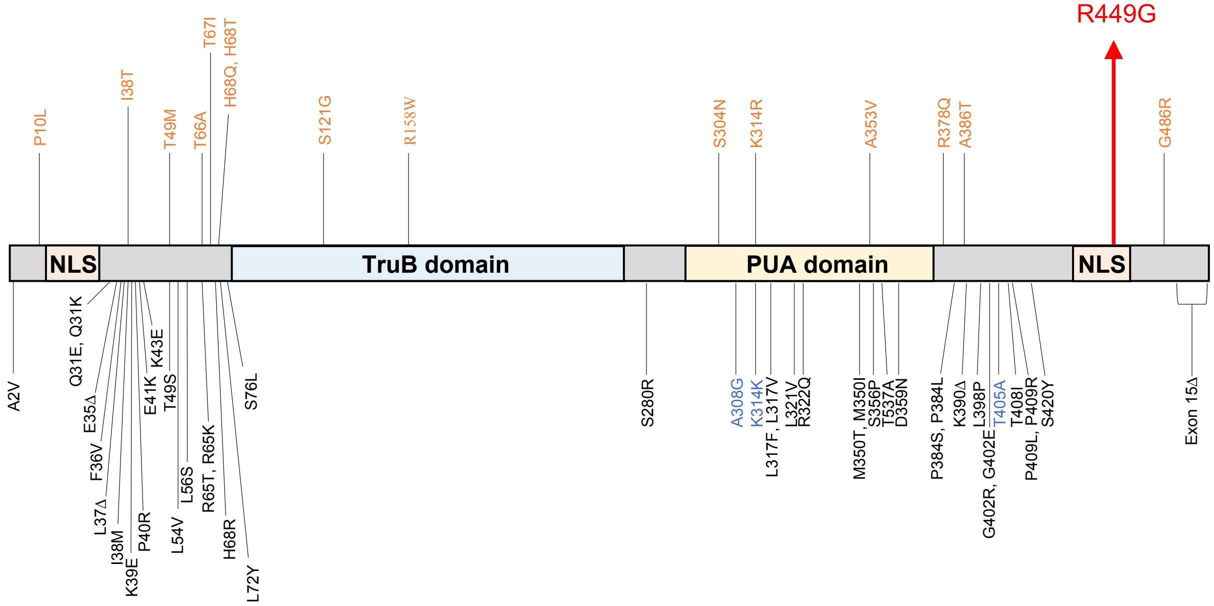

b

```
SP|O60832|DKC1_HUMAN   -VVAEAAKTAKRKRESESESDETPPAA------- 462
SP|Q9ESX5|DKC1_MOUSE   ----EAVNVIKRKRDSESESDETPTV-------- 461
SP|P40615|DKC1_RAT     -VTADAASIVKRKRDSDSDADEATPTT------- 463
TR|A7YWH5|A7YWH5_BOVIN -VVAEVVKTPKRKR--ESESEDTSPAA------- 460
SP|Q5ZJH9|DKC1_CHICK   -AVSEVERAPKRKRESESENEAVSPPPSPATPPP 463
TR|F7C3C0|F7C3C0_HORSE KVVAEAVKAPKRKR--ESESDETSPVS------- 470
                                :.   ****   :*: :  .
```

NLS

Supplementary figure 2. DKC1 domain and conservation in different species

a. Schematic diagram of the DKC1 protein with known domains, including the nuclear localization signal (NLS). The R449G mutation identified in this study is indicated with a red arrow. NLS: nuclear localization; TruB (PUS) domain: pseudouridine synthase domain; PUA domain: pseudouridine synthases & archaeosine-specific transglycosylases domain.

b. Conservative analysis shows a high degree of conservation of the arginine 449-containing portion in the DKC1 protein from other species. Arginine 449 is highlighted.

The red border indicates the nuclear localization signal.

Supplementary Figure 3

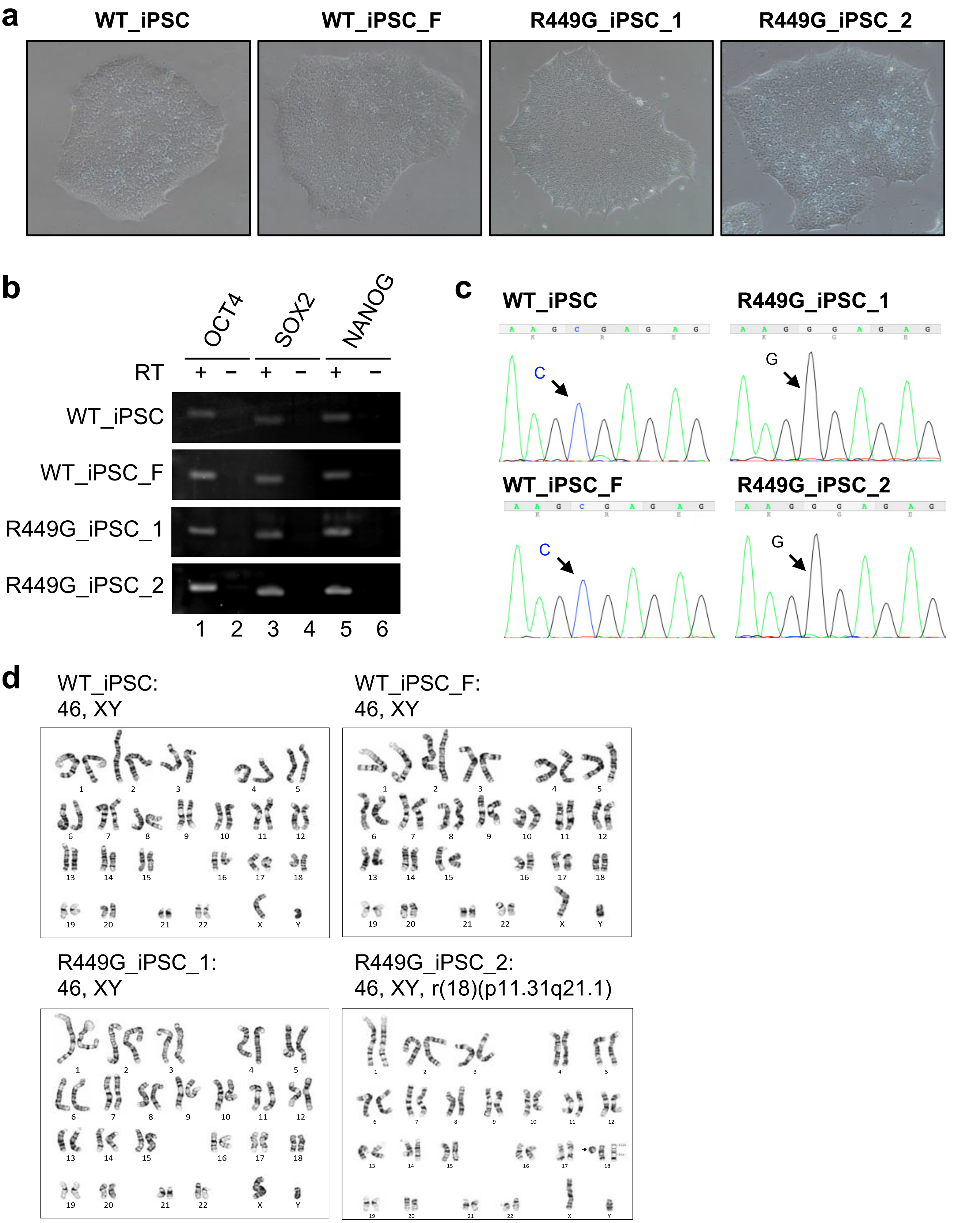

Supplementary figure 3. Derivation and characterization of DKC1 mutant iPSCs
a. Bright-field images of WT_iPSC, WT_iPSC_F, R449G_iPSC_1 and R449G_iPSC_2 cells. **b.** RT–PCR of pluripotency-associated gene expression in WT_iPSC, WT_iPSC_F, R449G_iPSC_1 and R449G_iPSC_2 cells. **c.** Sanger sequencing of codons 1342–1350 of WT_iPSC, WT_iPSC_F, R449G_iPSC_1 and R449G_iPSC_2 cells. c.1345C> G (p. R449G) mutation is indicated by the arrow. **d.** Metaphase G-banding karyotyping of iPSCs as indicated.

Supplementary Figure 4

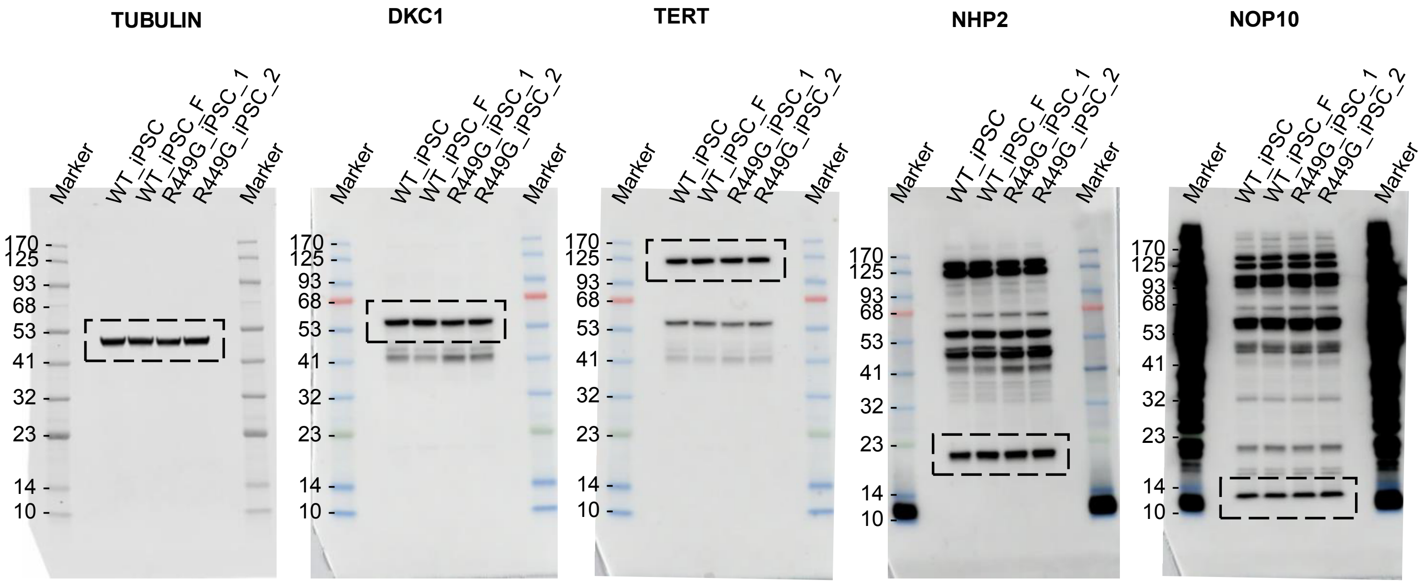

Supplementary Figure 4. Full blots of Figure 5b

Supplementary Figure 5

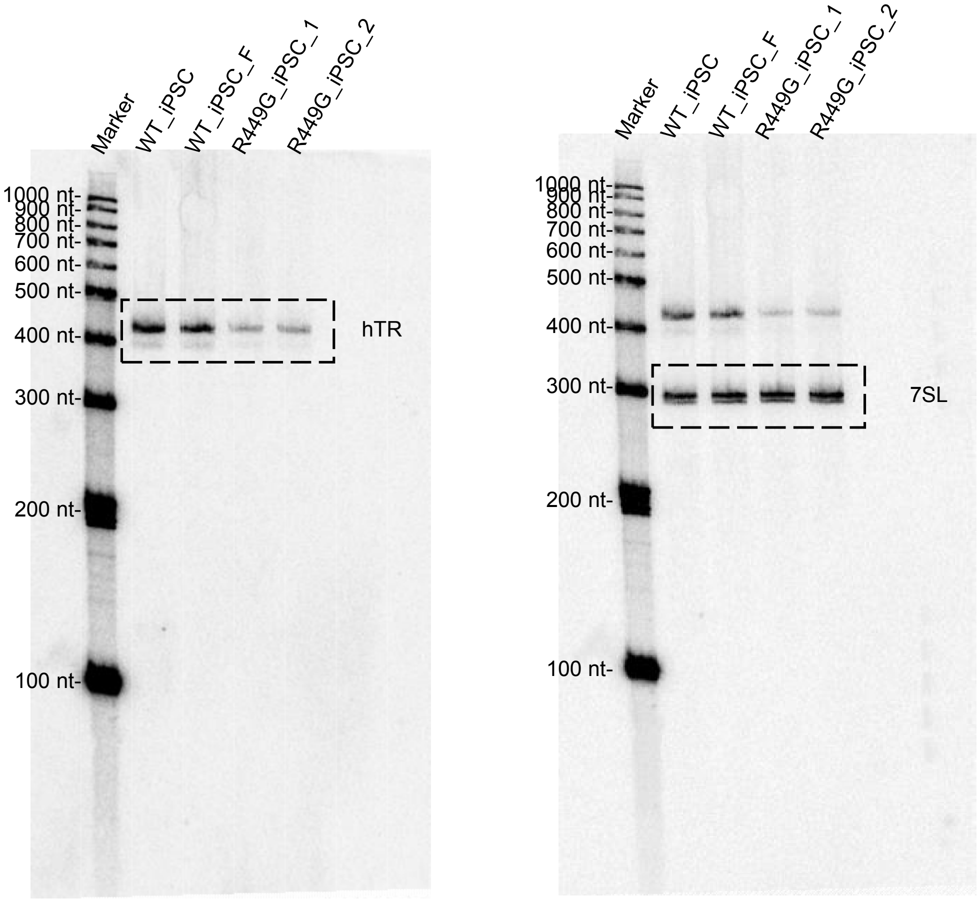

Supplementary Figure 5. Full blots of Figure 5c

Supplementary Figure 6

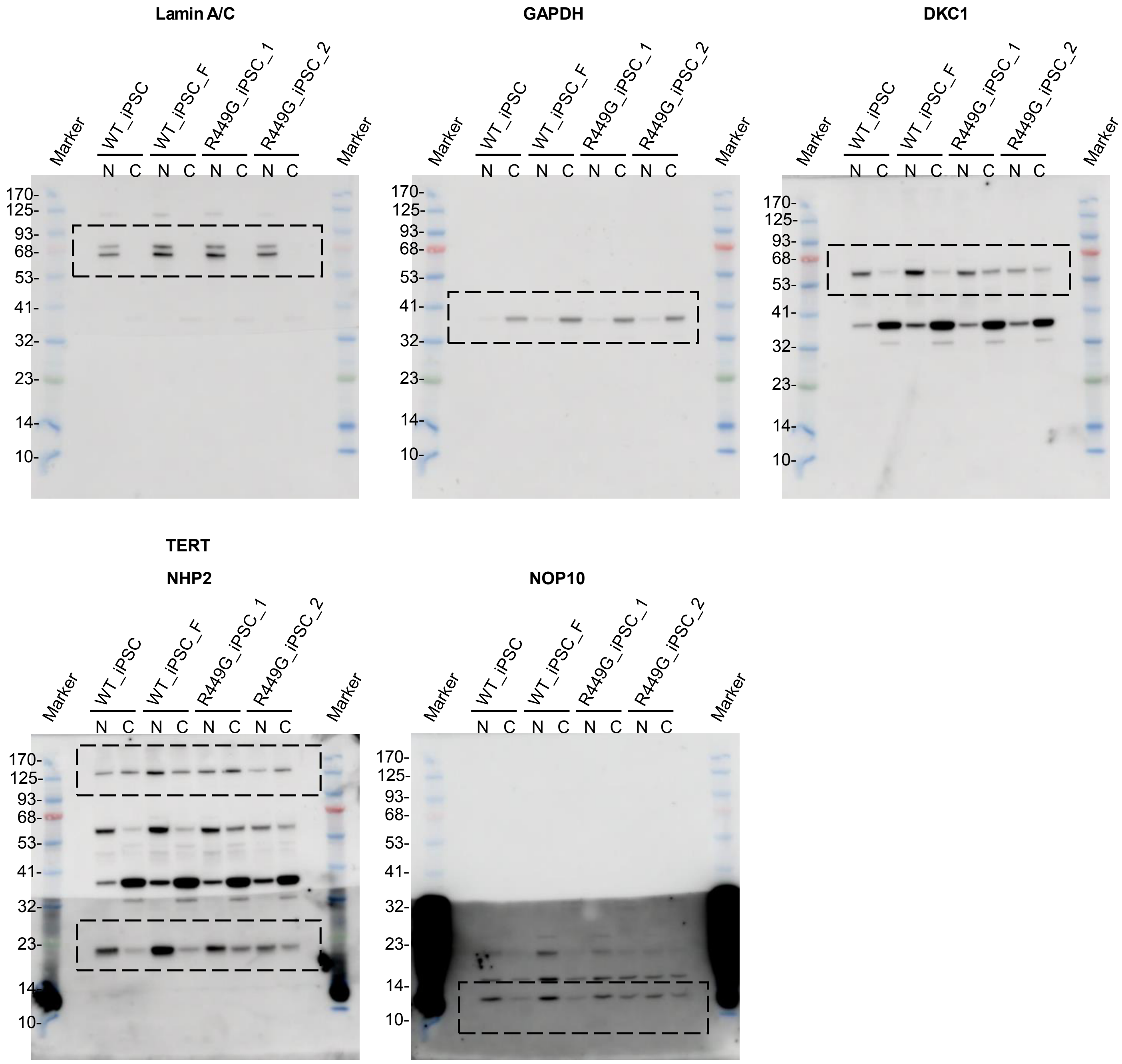

Supplementary Figure 6. Full blots of Figure 5e

Supplementary Figure 7

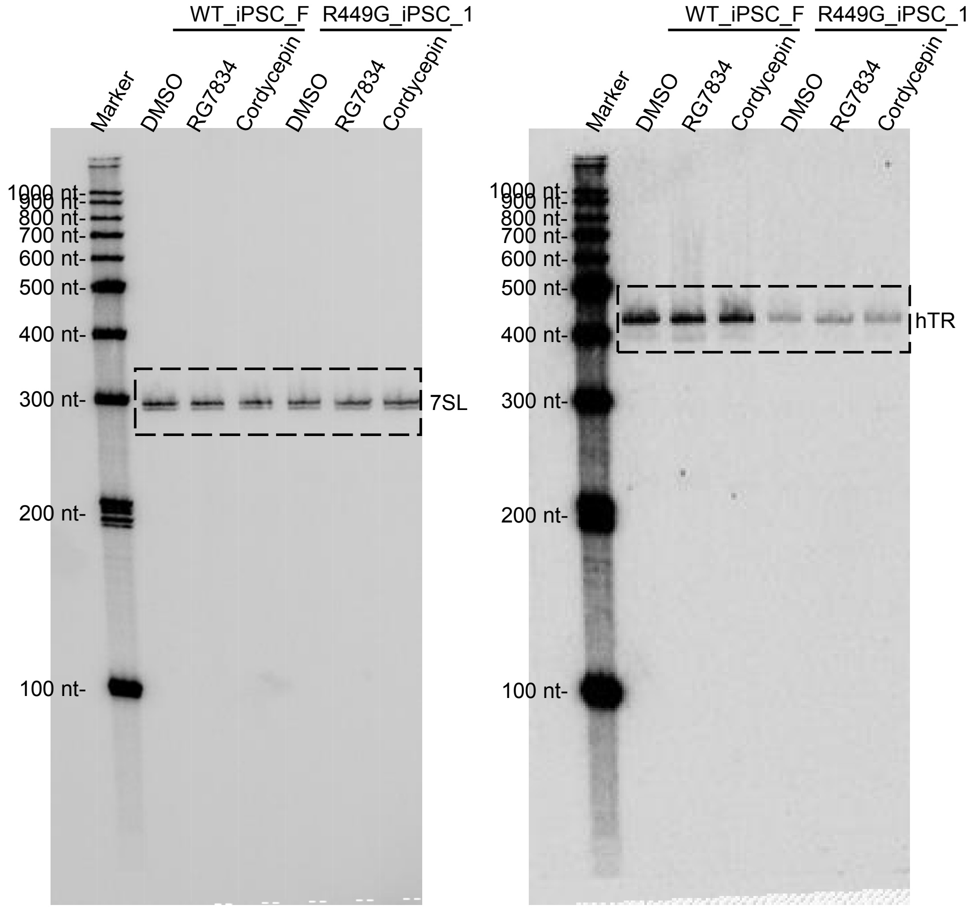

Supplementary Figure 7. Full blots of Figure 6a
