# Supplementary material for: A missense variant in the nuclear localization signal of DKC1 causes Hoyeraal-Hreidarsson syndrome
Source: NPJ Genom Med. 2022 Oct 30;7:64. doi: 10.1038/s41525-022-00335-8 (PMC9617742; doi:10.1038/s41525-022-00335-8)
Supplement: Supplementary file 2 — Reporting Summary [file 41525_2022_335_MOESM2_ESM.pdf]

nature research

Corresponding author(s): Chi-Kang Tseng

Reporting Summary

Nature Research wishes to improve the reproducibility of the work that we publish. This form provides structure for consistency and transparency in reporting. For further information on Nature Research policies, see Authors & Referees and the Editorial Policy Checklist.

Statistical parameters

When statistical analyses are reported, confirm that the following items are present in the relevant location (e.g. figure legend, table legend, main text, or Methods section).

n/a	Confirmed	
☐	☒	The exact sample size (*n*) for each experimental group/condition, given as a discrete number and unit of measurement
☐	☒	An indication of whether measurements were taken from distinct samples or whether the same sample was measured repeatedly
☐	☒	The statistical test(s) used AND whether they are one- or two-sided<br>*Only common tests should be described solely by name; describe more complex techniques in the Methods section.*
☐	☒	A description of all covariates tested
☐	☒	A description of any assumptions or corrections, such as tests of normality and adjustment for multiple comparisons
☐	☒	A full description of the statistics including central tendency (e.g. means) or other basic estimates (e.g. regression coefficient) AND variation (e.g. standard deviation) or associated estimates of uncertainty (e.g. confidence intervals)
☐	☒	For null hypothesis testing, the test statistic (e.g. *F*, *t*, *r*) with confidence intervals, effect sizes, degrees of freedom and *P* value noted<br>*Give P values as exact values whenever suitable.*
☒	☐	For Bayesian analysis, information on the choice of priors and Markov chain Monte Carlo settings
☐	☒	For hierarchical and complex designs, identification of the appropriate level for tests and full reporting of outcomes
☐	☒	Estimates of effect sizes (e.g. Cohen's *d*, Pearson's *r*), indicating how they were calculated
☐	☒	Clearly defined error bars<br>*State explicitly what error bars represent (e.g. SD, SE, CI)*

Our web collection on statistics for biologists may be useful.

Software and code

Policy information about availability of computer code

Data collection	The majority of software used in this study was also used in data analysis, but some software could be said to overlap with Data collection. For simplicity, we have listed all software used in the data analysis field.
Data analysis	Published software used are listed below. Specific usage details are given in the methods.<br><br>10X Loupe browser 6<br>10X CellRanger 4.0<br>Seurat v3.2.1<br>SeuratWrappers v0.3.0<br>SingleR v1.0.1

For manuscripts utilizing custom algorithms or software that are central to the research but not yet described in published literature, software must be made available to editors/reviewers upon request. We strongly encourage code deposition in a community repository (e.g. GitHub). See the Nature Research guidelines for submitting code & software for further information.

Data

Policy information about availability of data

All manuscripts must include a data availability statement. This statement should provide the following information, where applicable:

- Accession codes, unique identifiers, or web links for publicly available datasets
- A list of figures that have associated raw data
- A description of any restrictions on data availability

Sequencing datasets that support the findings of this study have been deposited to NCBI SRA database with the BioProject ID: PRJNA880366. Original data underlying gels and blots in this manuscript are given in Supplementary Figures.

Field-specific reporting

Please select the best fit for your research. If you are not sure, read the appropriate sections before making your selection.

☒ Life sciences ☐ Behavioural & social sciences ☐ Ecological, evolutionary & environmental sciences

For a reference copy of the document with all sections, see nature.com/authors/policies/ReportingSummary-flat.pdf

Life sciences study design

All studies must disclose on these points even when the disclosure is negative.

Sample size	No sample-size calculations were performed.
Data exclusions	Data were only excluded when contamination resulted in RNA degradation or technical problems (e.g. failed transfer from gel to membrane) prohibited data analysis.
Replication	All experiments were successfully replicated.
Randomization	No randomization
Blinding	Investigators were not blinded during experiments.

Reporting for specific materials, systems and methods

Materials & experimental systems

n/a	Involved in the study	
☐	☒	Unique biological materials
☐	☒	Antibodies
☐	☒	Eukaryotic cell lines
☒	☐	Palaeontology
☒	☐	Animals and other organisms
☒	☐	Human research participants

Methods

n/a	Involved in the study	
☒	☐	ChIP-seq
☒	☐	Flow cytometry
☒	☐	MRI-based neuroimaging

Unique biological materials

Policy information about availability of materials

Obtaining unique materials	No restrictions. Availability of commercially obtained antibodies in the future is out of our control

Antibodies

Antibodies used	Supplementary Table 7 and 8 provided with manuscript contains information on all antibodies used in the study
Validation	Supplementary Table 7 and 8 provided with manuscript contains information on all antibodies used in the study

Eukaryotic cell lines

Policy information about cell lines

Cell line source(s)	iPS cells, More information in Methods section.
Authentication	iPS cells, More information in Methods section.
Mycoplasma contamination	Negative for mycoplasma
Commonly misidentified lines (See ICLAC register)	None
